# Effects of Soil Chemical Factors on Leaf Traits and Fruit Quality of *Litsea mollis* Across Altitudinal Gradients

**DOI:** 10.3390/biology15131036

**Published:** 2026-06-29

**Authors:** Deng Wang, Luting Huang, Yeshe Wang, Shu Wang

**Affiliations:** 1 College of Agriculture, Forestry and Ecology, Shaoyang University, Shaoyang 422000, China; wangyeshe001@163.com; 2 College of Information Technology Application, Xiangzhong Normal College for Preschool Education, Shaoyang 422000, China; huanglutinggz@163.com; 3 School of Ecology, Northeast Forestry University, Harbin 230100, China; lnbx625@163.com

**Keywords:** *Litsea mollis*, altitude, nutrient, essential oil, citral

## Abstract

Despite the economic and medicinal value of the tree species *Litsea mollis* in southern China’s mountain forests, its wild populations remain understudied in terms of their adaptation to altitudinal gradients. This study examined *L. mollis* populations spanning altitudes of 760–1550 m in Nanshan Park, Hunan Province, to evaluate variation in leaf traits and fruit quality with elevation changes as well as associations with soil chemical properties. Results revealed that increasing altitude corresponded with higher leaf mass, chlorophyll content, soluble compound levels, enzyme activity, and various fruit quality traits (e.g., longitudinal and transverse diameters, weight, and fat, protein, carbohydrate, vitamin A, essential oil, and citral levels). Conversely, leaf area, specific leaf area, petiole length, fruit shape index, fruit stalk length, and ash content declined as altitude rose. Redundancy analysis indicated that specific leaf area, peroxidase activity, and Malondialdehyde content are the primary leaf characteristics influencing fruit quality, and soil pH and total nitrogen, alkaline nitrogen, and available potassium levels were key chemical factors shaping ecological adaptation and fruit quality of *L. mollis* along the altitudinal gradient. Overall, *L. mollis* augments light capture and nutrient acquisition by modifying morphological traits, such as leaf area (26.17%) and specific leaf area (44.32%), facilitating adaptation to low-light and nutrient-poor conditions at lower elevations. At higher elevations, plants preferentially allocate resources to increase leaf mass (33.33%) and chlorophyll content (19.02%), improving photosynthetic efficiency, osmotic regulation, and metabolic enzyme activity. This resource allocation promotes nutrient and secondary metabolite accumulation in fruit, enhancing plant stress resistance and fruit quality. This synergistic relationship represents an adaptive adjustment by *L. mollis* in allocating growth and reproductive resources across different altitude environments. These findings provide a theoretical framework for understanding altitudinal adaptation in *L. mollis* and offer practical guidance for its introduction, cultivation, and fruit quality improvement in high-elevation regions.

## Simple Summary

Despite the economic and medicinal values of *Litsea mollis* in South China’s mountain forests, its wild adaptive responses to altitudinal gradients remain poorly understood. This study investigated the effects of altitudinal gradients on leaf traits and fruit quality of *L. mollis*, and identified the key soil factors driving trait variation. Results showed that increasing altitude boosted leaf mass, chlorophyll, soluble substances, enzyme activity and fruit quality traits, while leaf area, specific leaf area and fruit ash content decreased. Core leaf and soil factors driving fruit quality were identified via redundancy analysis. At low elevations, the species expands leaf area to capture light and nutrients; at high elevations, it invests more biomass into leaves to strengthen photosynthesis and stress resistance, accumulating richer fruit metabolites. These findings clarify the altitudinal adaptation mechanism of *L. mollis*, and provide a theoretical basis for its cultivation, conservation and variety improvement.

## 1. Introduction

Altitude is a key geographic factor encompassing substantial variation in environmental elements, including temperature, light, humidity, soil fertility, and biological conditions [1]. Plant leaf and fruit traits exhibit adaptive shifts in response to altitudinal environments [2,3,4]. Currently, research on plant responses to high-altitude environments has primarily focused on individual leaf traits or fruit quality, with considerable variation observed among species. For example, *Schima superba* may decrease its leaf area and leaf mass, as well as specific leaf area, in response to environmental stress factors such as intense radiation and low temperatures at high altitudes. This reduction in photosynthetic area helps plants tolerate extreme conditions, whereas the accumulation of soluble sugars enhances stress resistance. Chlorophyll content exhibits a fluctuating pattern along altitudinal gradients, initially increasing and subsequently decreasing, to adapt to varying light intensities and temperatures across elevations [5]. Similarly, malondialdehyde content and antioxidant enzyme activities in the leaves of *Chenopodium quinoa* increase with altitude, indicating that high-altitude environments intensify oxidative stress and stimulate antioxidant defense systems to cope with adverse conditions [6]. However, several studies have reported that the physiological and morphological responses of leaves to altitude differ markedly among tree species and may even exhibit antithetical trends [7,8]. The mechanisms underlying this trait differentiation therefore require further investigation.

Altitudinal gradients strongly influence the nutritional composition, morphological characteristics, and flavor quality of tree fruit by altering photosynthetic accumulation, metabolic processes, and soil nutrient availability. However, the response patterns vary among species. In economic forest trees, suitable high-altitude environments can promote fruit quality improvement. Increasing altitude has been shown to enhance fruit development and seed quality in *Hippophae rhamnoides* and *Malus pumila* “Red Fuji” [9,10]. In *Juglans sigillata* from Yangbi, protein content increases whereas fat content decreases with altitude. In contrast, fruit horizontal diameter, per fruit weight, and pericarp thickness show no significant altitudinal response, highlighting the substantial plasticity of fruit quality traits [11]. Overall, *Prunus armeniaca* var. *ansu* has been found to exhibit decreased per fruit weight and increased carbohydrate accumulation, traits that are thought to reflect an adaptive resource allocation strategy used at high altitudes [4]. Turner et al. [12] verified that altitudinal variation markedly affects the flavor and quality of *Musa* × *paradisiaca* fruit.

Soil chemical factors, as key components of altitudinal gradients, directly and indirectly influence plant growth and development [13]. Essential soil nutrients, particularly nitrogen and phosphorus, are directly involved in photosynthesis, biomass accumulation, and organ formation. Soil nitrogen can promote leaf growth and chlorophyll synthesis, enhance photosynthetic efficiency, and provide the material basis for nutrient accumulation and fruit quality formation [14]. Soil phosphorus primarily regulates root development and energy metabolism. Adequate phosphorus availability promotes sugar synthesis and accumulation in fruit, thereby improving fruit flavor and quality [15]. Although the current research has clearly identified the independent effects of altitude and soil chemical properties on leaf and fruit traits, the synergistic regulatory effects of altitudinal gradients and soil chemical factors remain poorly understood. In addition, most studies have examined the altitudinal adaptability of leaf traits or fruit quality separately, without systematically evaluating the intrinsic relationships between leaf traits and fruit quality. Consequently, the ecological mechanisms underlying whole-plant adaptation to altitudinal environments remain incompletely understood, and the primary drivers of altitudinal variation in fruit quality have yet to be fully clarified.

Hunan Nanshan Park is situated in the summit region of the Nanling Mountain Range and exhibits a pronounced elevation gradient. *Litsea mollis* is a common tree species occurring in hillside scrub and broadleaved forests at altitudes of ~700–1600 m. The fruit of *L. mollis* is its primary source of essential oil, containing the highest concentrations. Its main component, citral, has antimicrobial, mosquito-repellent, insecticidal, and antioxidant properties [16,17,18], supporting its wide use in medical and food industries. Existing wild *L. mollis* populations are largely found in natural habitats, making it difficult to stabilize and control production and quality, with reliance on wild resources limiting the ability to meet market demand. Previous research on *L. mollis* has mainly focused on chemical composition [19], selection of superior plant or fruit phenotypes [20], variation in phenotypic traits and their dominant influencing factors [21], and fruit growth and development at different ripening stages [22]. However, the mechanisms underlying how wild germplasm responds to altitude in terms of leaf traits, fruit phenotypes, and quality remain unclear. Therefore, this study systematically analyzed *L. mollis* leaf trait and fruit quality responses to varying altitudes and their correlations with soil chemical factors in Nanshan Park, Hunan, China. This study comprehensively examined the responses of leaf traits and fruit quality of *L. mollis* to different altitudinal conditions and their relationships with soil chemical factors. The primary objectives were to investigate (1) variations in leaf traits and fruit quality across altitudinal gradients, (2) the relationships between leaf traits and fruit quality, and (3) the key soil chemical factors influencing leaf morphological traits and fruit quality.

## 2. Materials and Methods

### 2.1. Overview of the Study Area

Hunan Nanshan Park (26°01′–26°21′ N, 109°59′–110°33′ E) is located in the Nanling Mountain peaks at the intersection of east–west and north–south mountain systems in China, spanning evergreen broadleaved forest regions in the Yangtze River’s southern hilly basin and the Wuling Mountains. The region lies in the central subtropical zone and has a mountain-dominated subtropical monsoon humid climate. Elevation varies markedly from 425 to 1946 m. Vegetation is vertically zoned into subtropical evergreen broadleaved forest, evergreen–deciduous broadleaved forest, deciduous broadleaved forest, and mountain-top dwarf forest. The mean annual temperature is 16.1 °C, annual sunshine duration is 1138 h, the frost-free period is 271 days, relative humidity is 75–83%, and annual precipitation is 1100–1400 mm (mainly concentrated in May–June). Soils are primarily yellow-brown and classified as Podzoluvisols (according to the FAO soil classification) [5].

### 2.2. Experimental Materials

**Figure 1:**
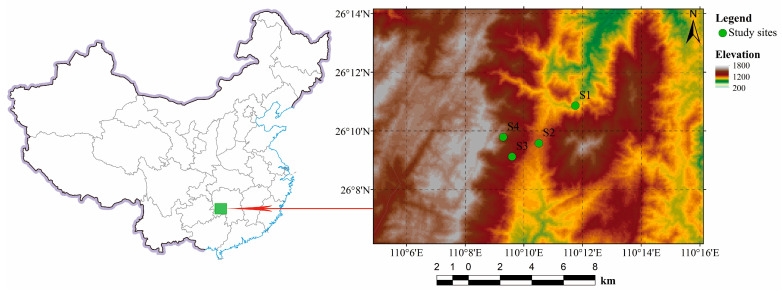
Map showing the study area and sampling plots.

Preliminary surveys identified sites dominated by *L. mollis*. Four plots (50 × 50 m) were established at ~200 m elevation intervals on the southern slopes of Nanshan National Park along an elevation gradient from 760 to 1550 m. The four plot elevations were 760, 1020, 1260, and 1550 m (Figure 1). In all plots, *L. mollis* individuals with heights of 2.5–3.2 m and diameter at breast height of 3.3–5.4 cm were present (Table 1). Sampling was conducted during October 2023. Five individuals were selected per sampling site, and 10–20 fully expanded sun leaves were collected from each tree. Four fruit clusters and 30 fruit were randomly collected from the upper, middle, lower, and inner canopy layers. During sampling, fruit stalks were retained to minimize loss of volatile compounds. Collected leaves, fruit, and fruit clusters were placed in labeled bags and stored in an insulated box at 4 °C. Soil samples (0–20 cm depth) beneath each tree were collected using a ring knife. After thorough mixing of soil samples from each altitude, ~2 kg subsamples were obtained by quartering and placed in labeled bags. Debris was removed and samples were air-dried, ground, sieved, and stored in sample bags for analysis. Soil chemical properties were measured from mixed samples at each altitude with 15 replicates. Results of detection and analysis are presented in Table 2.

### 2.3. Experimental Methods

#### 2.3.1. Leaf Characteristics

Leaf area (LA) was measured using a Yaxin-1241 leaf area meter (Beijing Yaxinliyi Science and Technology Co., Ltd., Beijing, China), and leaf petiole length (LPL) was measured using a vernier caliper (accuracy 0.01 mm). Leaf mass (LM) was determined after drying leaves at 70 °C to constant mass to obtain specific leaf area (SLA). To determine chlorophyll a and chlorophyll b contents, 10 mL of ice-cold 80% (*v*/*v*) acetone was used to extract these pigments from 0.05 g of fresh leaves. After mixing overnight and centrifuging for 10 min at 12,000 rpm, the supernatant was collected, and its absorbance was measured at 663, 646, and 470 nm. The total chlorophyll (Chl) was calculated using the equations described by Zou [23]. Soluble sugar content (SS) was determined using anthrone colorimetry [24]. About 0.5 g of fresh leaves were ground in liquid N_2_. The solution was taken in a test tube, 5 mL of distilled water was added, and the SS was extracted by placing it in boiling water for 30 min. After the solution cooled down to 25 °C, it was centrifuged at 5000 rpm for 5 min. The supernatant was poured into a 25 mL volumetric flask, and the extraction was repeated twice with constant volume. About 0.5 mL of the extract was taken in a hard test tube, and ~1.5 mL of distilled water, 0.5 mL of anthrone–ethyl acetate reagent, and 5 mL of concentrated sulfuric acid were added in an ice bath. After mixing, the solution was quickly heated in boiling water for 1 min, and the OD_630_ was measured. Malondialdehyde content (MDA) measured by the thiobarbituric acid method [24]. The samples were mixed with 0.5% thiobarbituric acid (TBA) prepared in 20% trichloroacetic acid (TCA) (or with 20% TCA without TBA for the controls); then, it was incubated at 95 °C for 20 min. After stopping the reaction on ice, the absorbance of the supernatants was measured at 532 nm. The nonspecific absorbance at 600 and 440 nm was subtracted. Activities of catalase (CAT), peroxidase (POD), and superoxide dismutase (SOD) were determined using commercial kits (Nanjing Jiancheng Bio-technology Co., Ltd., Nanjing, China).

#### 2.3.2. Fruit Phenotypic Traits

Each infructescence weight (IW) was weighed using an electronic balance. The number of drupes per cluster was recorded, and per fruit weight (PFW) was calculated as IW divided by drupe count. Using a vernier caliper, fruit longitudinal diameter (FLD), fruit horizontal diameter (FHD), and fruit stalk length (FSL) were measured individually in each fruit. The fruit shape index (FSI) was calculated as FLD/FHD. Seeds were manually separated, and their dimensions were measured using a vernier caliper. Fruit pericarp thickness (FPT) was calculated, and seed weight (SW) was determined using an electronic balance.

#### 2.3.3. Nutrient Composition and Volatile Compounds in Fruit

Moisture content (MC) was determined via direct drying (GB 5009.3—2016, Method 1) [25], ash content (AC) through calcination (GB 5009.4—2016) [26], fat content (FC) using the Soxhlet extraction method (GB 5009.6—2016, Method 1) [27], protein content (PC) via Kjeldahl nitrogen determination (GB 5009.5—2016, Method 1) [28], crude fiber content (CFC) according to GB 5009.10—2003 [29], carbohydrate content (Carb) following GB 28050—2011 [30], vitamin A content (VAC) through reversed-phase high-performance liquid chromatography (GB 5009.82—2016, Method 1) [31], and vitamin C content (VCC) using 2,6-dichloroindophenol titration (GB 5009.86—2016, Method 3) [32]. Essential oil content (EOC) was measured via steam distillation (GB 11424-2008) [33], with essential oil components analyzed using capillary column gas chromatography following GB/T 11424-2008. Citral content (CC) in essential oil was determined using area normalization [33].

### 2.4. Data Analysis

All data were analyzed using SPSS 18.0 (IBM, Armonk, NY, USA). Data normality and homogeneity of variance were assessed using the Shapiro–Wilk and Bartlett tests, respectively. One-way ANOVA followed by Tukey’s honestly significant difference test was employed to compare differences in *L. mollis* leaf traits and fruit quality (phenotype traits, nutrient content, and volatile compounds) across altitudes (*p* < 0.05). For each variable, 15 biological replicates were used per altitude (5 plants × 3 replicates), and results are represented as means ± standard errors. RDA was conducted using Canoco 5.0 (Microcomputer Power, Ithaca, NY, USA) to evaluate relationships among soil chemical factors, leaf traits, and fruit quality in *L. mollis*. Data were standardized, and Monte Carlo permutation tests were used to assess the significance of leaf traits on fruit quality, and soil chemical factors on leaf traits and fruit quality, respectively [34].

## 3. Results

### 3.1. Leaf Morphology and Physiological Indicators

Except for LA, SLA, and LPL, other leaf traits exhibited an increasing trend with altitude (Table 3). Specifically, LA showed a significant decline with increasing altitude (*p* < 0.05), SLA increased significantly at 1020 m (*p* < 0.05), whereas LPL exhibited significant variation at 1260 m (*p* < 0.05). LM, MDA, SOD, and CAT showed no significant differences between 760 and 1020 m. These traits increased significantly only above 1260 m (*p* < 0.05), with subsequent changes remaining nonsignificant. Chl and SS levels did not differ significantly between 760 and 1260 m but became significant at 1550 m (*p* < 0.05). POD remained stable at 1020 and 1260 m but significantly increased at 1550 m (*p* < 0.05).

### 3.2. Fruit Phenotype and Nutrients

Except for FSI and FSL, other fruit phenotypic traits increased with altitude (Table 4). Significant differences (*p* < 0.05) were observed in FLD, FHD, FPT, SW, and FSL across altitudes. The FSI significantly decreased only at 1260 m (*p* < 0.05), after which no further significant changes occurred. The difference in PFW between 1260 and 1550 m was nonsignificant; however, significant differences were observed at other elevations (*p* < 0.05). Additionally, IW did not differ significantly between 1020 and 1260 m but was significantly greater at 760 m (*p* < 0.05).

Significant differences in MC, FC, and EOC were observed across altitudes (*p* < 0.05, Table 5). PC significantly increased from 760 to 1260 m (*p* < 0.05), after which no further significant changes occurred. Carb significantly increased only at 1550 m (*p* < 0.05). VAC significantly increased at 1020 m (*p* < 0.05), although subsequent increases were nonsignificant. CC significantly increased at 1020 m, showed no significant change between 1020 and 1260 m, and increased significantly at 1550 m (*p* < 0.05). Altitude had no significant effect on CFC or VCC. AC showed no significant difference between 760 and 1020 m but decreased significantly at 1260 m (*p* < 0.05).

### 3.3. Cross-Altitude Relationships Among Leaf Traits, and Fruit Quality in L. mollis Across Altitudes

**Figure 2:**
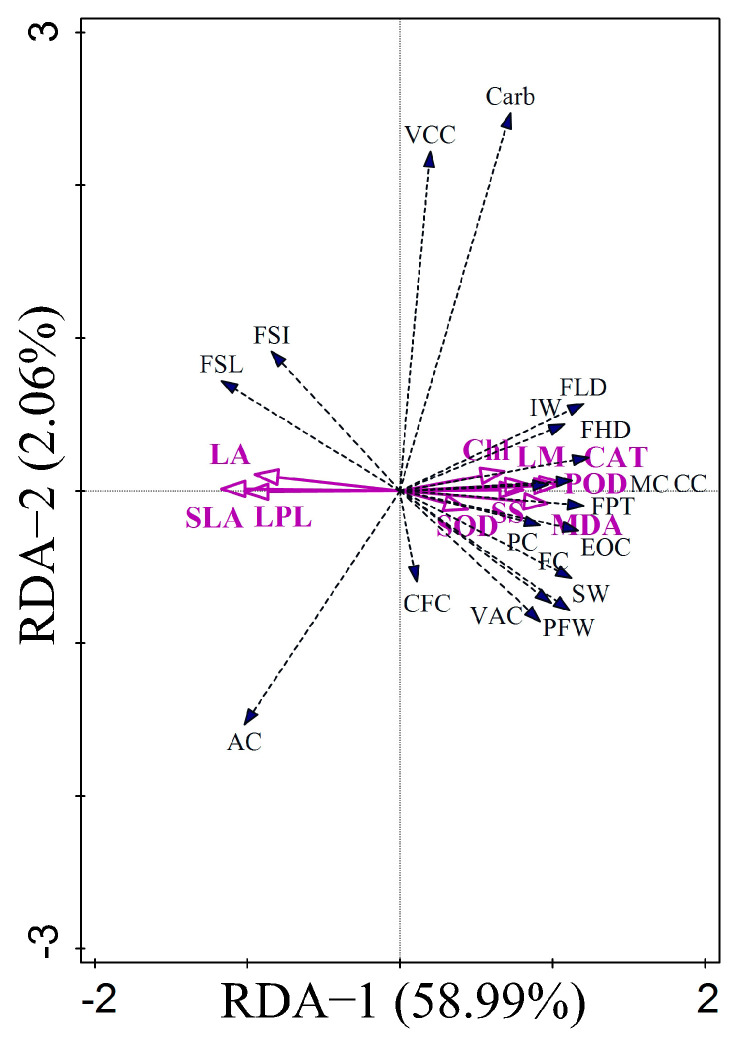
Redundancy analysis of leaf and fruit quality traits across altitudes.

The relationships between 10 leaf and 18 fruit quality traits of *L. mollis* were analyzed using redundancy analysis (RDA; Figure 2). The first axis (horizontal axis) explained 58.99% of the total variation, whereas the second axis (vertical axis) explained 2.06%. LA, SLA, LPL, PSI, and PSL showed significant positive correlations. In addition, the remaining leaf traits exhibited strong positive correlations with EOC, PC, FC, SW, PFW, VAC, MC, CC, FPT, IW, FHD, and FLD. SLA (41.6%), POD activity (9.20%), MDA content (4.60%), LPL (3.10%), Chl content (1.80%), and LM (1.40%) were identified as the primary factors contributing to variation in fruit quality of *L. mollis* (Table 6).

### 3.4. Relationship Among Soil Chemical Factors, Leaf Traits, and Fruit Quality in L. mollis Across Altitudes

**Figure 3:**
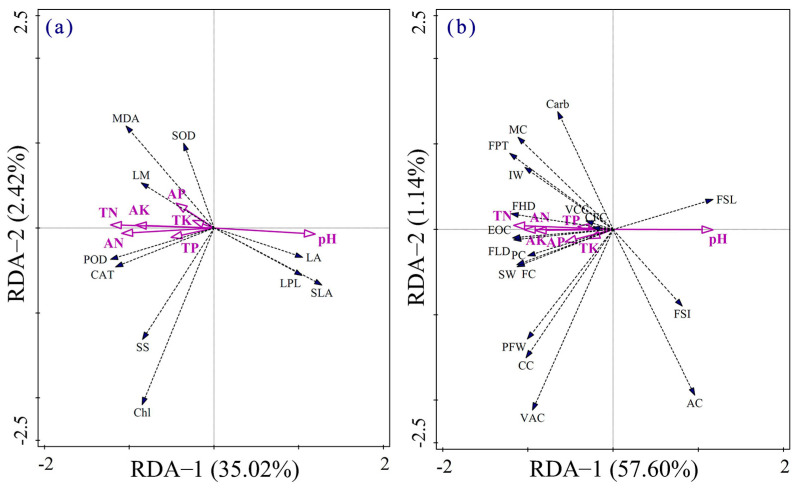
Redundancy analysis of soil chemical factors and leaf traits (**a**) and fruit quality traits (**b**) of *L. mollis* across altitudes.

The relationships among 10 leaf traits of *L. mollis* and soil chemical factors were analyzed using RDA analysis (Figure 3a). The horizontal axis representing the first-order axis accounted for 35.02% of the variation, whereas the vertical axis representing the second-order axis accounted for 2.42%. Notably, LA, SLA, LPL, and soil pH showed significant positive correlations. Additionally, Chl, SS content, CAT activity, POD activity, LM, MDA content, and SOD activity exhibited strong positive correlations with soil AK, TN, and AN. Soil TN (26.00%) was identified as the primary factor influencing variation in leaf traits of *L. mollis*, followed by AN (6.40%), pH (3.50%), and AK (2.30%; Table 7). RDA analysis of 18 fruit quality indicators (Figure 3b) revealed that the horizontal axis explained 57.60% of variation, whereas the vertical axis explained 1.14%. Significant positive correlations were observed among FSL, FSI, AC, and soil pH. The remaining indicators demonstrated significant positive correlations with soil TN, TP, AN, and AK. Soil pH (42.30%) was the primary factor explaining fruit quality variation, followed by AK (9.00%), AN (3.80%), and TN (3.00%; Table 7).

## 4. Discussion

Leaves are the primary organs through which plants interact with the external environment and are highly sensitive to environmental change. They directly participate in photosynthesis, carbon sequestration, respiration, and transpiration [35]. This study reveals that as altitude increases, LM in *L. mollis* also increases, whereas LA and SLA decline. This finding aligns with the observations reported by Xiang [36]. At higher altitudes, plants typically reduce transpiration, mitigate intense radiation, and avoid damage from low temperatures and strong winds by decreasing LA and SLA [5]. Conversely, Tu [37] reported that the SLA of *L. elongata* in the Dabie Mountains remained relatively stable across 592–1200 m. This discrepancy may reflect different adaptative strategies among *Litsea* species under specific altitudinal conditions. Higher LM suggests stronger structural support and defense, indicating more resilient leaves [38]. Moreover, the functional importance of the petiole is frequently underestimated relative to other leaf traits. Our results indicate that LPL in *L. mollis* diminishes progressively with increasing altitude, consistent with the findings of Ma et al. [39]. Shorter LPL positions leaves closer to branches, enhancing structural support and protection while reducing susceptibility to damage from external forces such as strong winds. This conservative strategy effectively minimizes energy loss [40]. Additionally, Chl, SS, and MDA content, along with POD and CAT activity, in *L. mollis* all increased with altitude. As altitude rises and light intensity increases, plants absorb more short- and long-wave radiation, improving light energy transfer and promoting photosynthetic pigment biosynthesis. This adjustment is crucial for coping with lower temperatures and shorter growing seasons at high elevations [41]. High-altitude stressors, including low temperature, drought, and intense radiation, promote accumulation of MDA, a product of membrane lipid peroxidation, in plant cells. In response, antioxidant enzyme systems are activated, mitigating membrane damage by upregulating CAT and POD activities and enhancing self-protection [42,43]. Concurrently, elevated SS concentrations help prevent cellular dehydration, maintain membrane and enzyme stability, enhance stress tolerance, and support normal metabolism [44].

With increasing altitude, FLD, FHD, PFW, IW, FPT, and SW in *L. mollis* gradually increased, whereas FSI and FSL showed opposite trends. This pattern suggests that fruit morphology becomes more robust, with larger size and thicker pericarp improving seed protection and tolerance to harsh environments [45]. These findings align with the those of Yang et al. [46] and Guo [12]. However, studies on *Vaccinium myrtillus* [47] and *Vitis vinifera* [48] reported opposite trends, indicating that increased altitude imposes stress that limits fruit growth and nutrient accumulation, reducing fruit size. The increase in PFW and IW may reflect a reproductive strategy in which plants allocate more resources to fewer fruit under limited-resource conditions, thereby enhancing offspring survival. This pattern is consistent with findings by Zhang et al. [49] on the Para Yingda mango and Yang et al. [46] on *Torreya grandis*. Our results show that the FSI and FSL of *L. mollis* decline with increasing altitude, consistent with the findings of Shi et al. [50] for *H. rhamnoides* populations at 860–1810 m. Reduced FSI may reflect more compact morphology, which limits water loss and heat dissipation while reducing stress at the fruit–branch junction, thereby lowering the likelihood of fruit shedding. In contrast, studies by Xi et al. [51] on late-maturing navel orange varieties in Fengjie and by Gong [52] on *Citrus reticulata* × *sinensis* reported inconsistent patterns. Shorter fruit stems position fruit closer to the plant, improving support and protection while reducing shedding due to strong winds or mechanical damage. However, Xu et al. [53] suggested that longer stalks can better withstand bending stress and improve stability; these discrepancies likely reflect differences in altitude, species-specific traits, response mechanisms, and local climate conditions, among other factors.

Nutrient content, including FC, PC, Carb, VAC, EOC, CC, and other volatile compounds, increased significantly with altitude in *L. mollis* fruit. Thus, *L. mollis* appears to achieve heightened fruit quality under high-altitude conditions, with optimal values at 1550 m. Increased FC and PC levels may reflect enhanced nutrient reserves for seeds, helping plants cope with harsh conditions and extended germination periods at high elevations. Yang et al. [54] reported that PC in *J. sigillata* increases with altitude, whereas FC decreases. Such differences may result from species-specific metabolic pathways or varying environmental regulation of nutrient synthesis. Carbs help maintain osmotic balance and metabolic stability, serving as protective compounds against reactive oxygen species in plants [55]. Higher carbohydrate levels may improve the survival of *L. mollis* under low-temperature conditions [56]. Vitamin A and its derivatives, as carotenoid precursors, play key roles in scavenging free radicals and reducing oxidative stress [57]. Under high-altitude conditions of low temperature and oxygen, seeds may prioritize stress resistance and nutrient supply by reallocating photosynthates toward carbohydrates and antioxidants. Metabolic regulation may achieve coordinated adaptation by enhancing enzyme activity, carbon metabolism, and carotenoid biosynthesis. Essential oils and citral, key secondary metabolites in *L. mollis*, contribute to fruit flavor and aroma while also participating in stress-related signaling pathways. Their increased accumulation likely enhances plant resistance to biotic and abiotic stress. Previous studies indicated that strong radiation, low temperature, and large diurnal temperature variation at high altitudes can stimulate production of secondary metabolites [58,59]. Together, these changes represent the adaptive strategies of *L. mollis* to high-altitude environments.

Our research also revealed notable positive correlations among LA, SLA, LPL, PSI, and PSL in *L. mollis*. In addition, other leaf traits were positively correlated with EOC, PC, FC, SW, PFW, VAC, MC, CC, FPT, IW, FHD, and FLD. These results indicate that variation in leaf functional traits can synergistically influence fruit development and quality. Such trait associations reflect the coordinated adaptive responses of *L. mollis* to changes in altitudinal environments. Individuals with larger leaf area and specific leaf area generally possess a greater capacity for light interception, providing more carbohydrates and energy for fruit development and thereby promoting increases in PSI [60]. The positive correlations among LPL, PSL, and PSI may reflect coordinated development of the vascular transport system, as longer petioles are often associated with more developed transport tissues that facilitate the efficient translocation of photosynthetic products to fruit.

As key components of the antioxidant defense system, elevated POD and CAT activities can more effectively scavenge reactive oxygen species, maintain cell membrane integrity, and preserve photosynthetic stability, thereby supporting the continuous production and transport of assimilates. This process is closely associated with the accumulation of secondary metabolites, including essential oils and vitamin C, in fruit [61]. Soluble sugar and chlorophyll contents directly reflect leaf photosynthetic capacity and carbon nutritional status. Higher soluble sugar concentrations in leaves provide a more abundant carbon source for the synthesis of storage compounds such as fats and proteins in fruit [62]. Although MDA content is generally considered an indicator of membrane lipid peroxidation, a moderate increase may also be associated with enhanced metabolic activity, reflecting the accumulation of metabolic by-products under conditions of high photosynthetic activity.

The present study also showed that soil pH, TN, AN, and AK are key factors explaining variation in *L. mollis* leaf traits and fruit quality. Soil pH is strongly positively correlated with morphological traits, including LA, SLA, LPL, FSL, FSI, and AC. Soil pH influences plant growth by regulating nutrient availability and microbial activity, inducing species-specific responses [13]. In more acidic soils, smaller LA and SLA help reduce nutrient and water loss and improve resource-use efficiency under acidic stress [63]. In soils with higher pH, enhanced microbial activity promotes organic matter decomposition and nutrient release, supporting fruit development while improving morphology and quality. Soil nutrients, including TN, TP, AN, and AK, are closely associated with multiple leaf physiological and fruit quality traits of *L. mollis*. Thus, the combined effects of soil nitrogen, phosphorus, and potassium appear to strongly affect leaf physiology while promoting nutrient accumulation and secondary metabolite synthesis in *L. mollis*. Soil TN and AN are major nitrogen sources for plants. Nitrogen is a fundamental component of enzymes and chloroplasts. It contributes to photosynthetic pigment synthesis and protein formation [14] while regulating fruit development through effects on cell division and expansion. This dual role explains its contribution to increased LM and PFW. Phosphorus promotes sugar conversion in plants, supports starch, protein, and fat synthesis, and participates in carbohydrate, nitrogen, and lipid metabolism. Under low temperatures, AK enhances stress resistance by regulating osmotic balance, facilitating sugar transport, and increasing antioxidant enzyme activity [64].

Although this study provides a comprehensive analysis of the ecological adaptation mechanisms of *L*. *mollis* across altitudinal gradients, several limitations remain. First, environmental influences are inherently complex and interactive; however, the present study primarily focused on the effects of soil chemical properties on leaf traits and fruit quality and did not consider other important factors such as seasonal variations in temperature, humidity, and light intensity or biological influences such as pollinators and symbiotic microorganisms. Collectively, these factors may regulate the growth, development, and adaptive strategies of *L*. *mollis*. Future studies should incorporate long-term fixed-site observations to examine the interactions among multiple environmental variables and further elucidate the molecular mechanisms underlying altitudinal responses. Such approaches would provide a stronger theoretical basis for the conservation, sustainable utilization, and varietal improvement of this species.

## 5. Conclusions

In summary, altitudinal variation significantly affected *L. mollis* leaf traits and fruit quality. With increasing altitude, LA, SLA, and LPL decreased, whereas Chl, osmotic substance levels, and antioxidant enzyme activity increased. Additionally, fruit phenotype, along with nutrient and volatile compound content, exhibited improved quality traits. After adapting their morphological and physiological traits to high-altitude stress conditions, leaves enhance photosynthetic capacity and stress tolerance, which directly promote fruit development and nutrient accumulation in *L. mollis*. This coordinated relationship represents an adaptive adjustment in resource allocation between growth and reproduction along altitudinal gradients, thereby maintaining the reproductive fitness of *L. mollis* under high-altitude conditions. Specific leaf area, POD activity, and MDA content were found to be key drivers of variation in leaf traits, and soil pH and total nitrogen, alkaline nitrogen, and available potassium levels were key chemical factors shaping ecological adaptation and fruit quality of *L. mollis* along the altitudinal gradient. These findings clarify the ecological adaptation mechanisms of *L. mollis* under varying altitude conditions. Furthermore, for cultivation of *L. mollis* in regions with pronounced elevation gradients, soil chemical properties and associated variation in leaf traits and fruit quality should be considered to optimize growth and yield.

## Figures and Tables

**Table 1:** Study site information of *Litsea mollis*.

Site Name	Elevation (m)	Latitude and Longitude	Plant Height (m)	Diameter at Breast Height (cm)
S1	760	110°11′45″ E, 26°10′52″ N	3.2 ± 0.4	5.4 ± 0.4
S2	1020	110°10′31″ E, 26°9′35″ N	3.0 ± 0.5	4.8 ± 0.7
S3	1260	110°9′36″ E, 26°9′7″ N	2.6 ± 0.2	3.7 ± 0.5
S4	1550	110°9′ 18″ E, 26°9′47″ N	2.5 ± 0.3	3.3 ± 0.3

**Table 2:** Chemical properties of soil beneath *L. mollis* trees across altitudes.

Altitude/m	Total Nitrogen Content (TN)/(g·kg^−1^)	Alkaline Nitrogen Content (AN)/(mg·kg^−1^)	Total Phosphorus Content (TP)/(g·kg^−1^)	Available Phosphorus Content (AP)/(mg·kg^−1^)	Total Potassium Content (TK)/(g·kg^−1^)	Available Potassium Content (AK)/(mg·kg^−1^)	pH
760	1.95 ± 0.20 c	95.44 ± 16.10 c	0.73 ± 0.10 a	2.55 ± 0.21 c	6.98 ± 0.40 b	136.15 ± 17.82 b	5.66 ± 0.32 a
1020	2.11 ± 0.34 c	121.75 ± 16.35 b	0.76 ± 0.09 a	2.77 ± 0.27 b	7.25 ± 0.60 ab	144.10 ± 16.65 b	5.25 ± 0.32 b
1260	2.45 ± 0.22 b	128.14 ± 19.22 b	0.76 ± 0.08 a	2.98 ± 0.19 a	7.72 ± 0.90 a	170.48 ± 14.18 a	5.02 ± 0.19 b
1550	2.81 ± 0.12 a	148.50 ± 24.34 a	0.80 ± 0.07 a	2.76 ± 0.20 b	7.13 ± 0.71 ab	165.61 ± 10.94 a	4.68 ± 0.24 c

Note: Different lowercase letters in the same column indicate significant differences (*p* < 0.05).

**Table 3:** Leaf traits of *L. mollis* at different altitudes.

Traits	Altitude/m			
	760	1020	1260	1550
LA cm^2^	31.87 ± 2.79 a	28.91 ± 5.40 b	24.30 ± 3.89 c	23.53 ± 2.80 d
LM g	0.12 ± 0.01 c	0.13 ± 0.03 bc	0.15 ± 0.03 ab	0.16 ± 0.01 a
SLA cm^2^·g^−1^	256.84 ± 40.01 a	222.82 ± 44.17 b	162.24 ± 15.30 c	143.02 ± 17.56 c
LPL cm	1.46 ± 0.15 a	1.32 ± 0.19 ab	1.20 ± 0.20 bc	1.05 ± 0.11 c
Chl mg·g^−1^	3.05 ± 0.33 b	3.28 ± 0.43 ab	3.26 ± 0.35 ab	3.63 ± 0.38 a
SS mg·g^−1^	5.97 ± 0.46 b	6.22 ± 0.62 b	6.42 ± 0.61 b	7.00 ± 0.56 a
MDA nmol·g^−1^	14.10 ± 1.67 c	15.74 ± 1.30 bc	16.89 ± 1.76 ab	17.93 ± 2.13 a
SOD U·g^−1^	12.10 ±1.26 b	13.27 ± 1.06 ab	13.53 ± 1.48 a	13.16 ± 1.62 ab
POD U·mg^−1^	8.43 ± 0.89 c	9.75 ± 0.90 b	9.84 ± 1.12 b	11.95 ± 1.44 a
CAT mg·g^−1^·min^−1^	5.58 ± 1.00 b	5.72 ± 1.30 b	7.55 ± 1.36 a	8.68 ± 1.35 a

Note: Different lowercase letters in the same row indicate significant differences (*p* < 0.05).

**Table 4:** Fruit phenotypic traits of *L. mollis* at altitudinal gradients.

Traits	Altitude/m			
	760	1020	1260	1550
FLD mm	5.28 ± 0.10 d	5.59 ± 0.12 c	5.88 ± 0.17 b	6.21 ± 0.11 a
FHD mm	5.05 ± 0.07 d	5.40 ± 0.13 c	5.83 ± 0.16 b	6.19 ± 0.08 a
FSI	1.05 ± 0.02 a	1.03 ± 0.02 a	1.01 ± 0.02 b	1.00 ± 0.02 b
FSL mm	6.07 ± 0.15 a	5.65 ± 0.09 b	5.90 ± 0.07 c	5.13 ± 0.06 d
PFW g	0.16 ± 0.01 c	0.19 ± 0.01 b	0.21 ± 0.01 a	0.21 ± 0.01 a
IW g	0.31 ± 0.02 c	0.38 ± 0.03 b	0.38 ± 0.02 b	0.47 ± 0.02 a
FPT cm	0.34 ± 0.01 d	0.39 ± 0.01 c	0.43 ± 0.01 b	0.47 ± 0.02 a
SW g	0.042 ± 0.003 d	0.048 ± 0.002 c	0.055 ± 0.003 b	0.061 ± 0.002 a

Note: Different lowercase letters in the same row indicate significant differences (*p* < 0.05).

**Table 5:** Fruit quality traits of *L. mollis* across altitudes.

Traits	Altitude/m			
	760	1020	1260	1550
MC g·100 g^−1^	54.11 ± 1.82 d	58.39 ± 1.43 c	61.35 ± 1.67 b	65.82 ± 2.61 a
AC g·100 g^−1^	1.33 ± 0.07 a	1.36 ± 0.07 a	1.20 ± 0.07 b	1.05 ± 0.08 c
FC g·100 g^−1^	10.33 ± 0.76 d	13.17 ± 1.19 c	15.36 ± 0.94 b	16.09 ± 0.58 a
PC g·100 g^−1^	3.97 ± 0.14 c	4.38 ± 0.09 b	4.50 ± 0.17 a	4.55 ± 0.15 a
CFC g·100 g^−1^	8.17 ± 0.15 a	8.12 ± 0.34 a	8.27 ± 0.51 a	8.33 ± 0.63 a
Carb g·100 g^−1^	1.85 ± 0.26 b	1.97 ± 0.18 b	1.92 ± 0.19 b	2.28 ± 0.12 a
VAC μg·100 g^−1^	339.89 ± 18.56 b	408.06 ± 14.15 a	410.05 ± 20.55 a	418.09 ± 19.01 a
VCC mg·100 g^−1^	15.55 ± 1.96 a	15.49 ± 1.77 a	16.18 ± 1.46 a	16.22 ± 2.35 a
EOC %	3.53 ± 0.16 d	4.07 ± 0.06 c	4.31 ± 0.11 b	4.65 ± 0.17 a
CC %	59.35 ± 4.98 c	73.33 ± 0.92 b	73.73 ± 0.68 b	77.51 ± 5.78 a

Note: Different lowercase letters in the same row indicate significant differences (*p* < 0.05).

**Table 6:** Variation in dimensions of *L. mollis* fruit quality explained by individual leaf traits.

Traits	Explains %	Contribution %	*F* Value	*p*
SLA	41.6	64.4	41.3	0.002
POD	9.2	14.3	10.7	0.002
MDA	4.6	7.2	5.8	0.002
LPL	3.1	4.8	4.1	0.002
Chl	1.8	2.7	2.4	<0.05
LM	1.4	2.2	1.9	<0.05
CAT	1	1.5	1.3	>0.05
LA	0.8	1.3	1.1	>0.05
SOD	0.6	1	0.9	>0.05
SS	0.5	0.7	0.6	>0.05

**Table 7:** Proportions of total variation in leaf and fruit quality traits of *L. mollis* explained by individual environmental factors.

Soil Chemical Properties	Leaf Trait				Fruit Quality			
	Explains/%	Contribution/%	*F* Value	*p*	Explains/%	Contribution/%	*F* Value	*p*
TN	26.00	61.600	20.400	<0.001	3.00	4.90	3.900	0.002
AN	6.40	15.100	5.400	<0.001	3.80	6.10	4.700	0.002
pH	3.50	8.200	3.000	<0.001	42.30	69.20	42.500	0.002
AK	2.30	5.500	2.100	0.05	9.00	14.80	10.600	0.002
AP	2.00	4.800	1.800	>0.05	1.10	1.80	1.500	>0.05
TP	1.50	3.700	1.400	>0.05	0.70	1.10	0.900	>0.05
TK	0.50	1.200	0.500	>0.05	1.30	2.10	1.700	>0.05

## Data Availability

The data presented in this study are available upon request from the corresponding author.

## Abbreviations

The following abbreviations are used in this manuscript:

LA	Leaf area	SW	Seed weight
LPL	Leaf petiole length	MC	Moisture content
LM	Leaf mass	AC	Ash content
SLA	Specific leaf area	FC	Fat content
Chl	Chlorophyll content	PC	Protein content
SS	Soluble sugars content	CFC	Crude fiber content
MDA	Malondialdehyde content	Carb	Carbohydrate content
POD	Peroxidase activity	VAC	Vitamin A content
SOD	Superoxide dismutase activity	VCC	Vitamin C content
CAT	Catalase activity	EOC	Essential oil content
IW	Infructescence weight	CC	Citral content
PFW	Per fruit weight	TN	Total nitrogen content
FLD	Fruit longitudinal diameter	AN	Alkaline nitrogen content
FHD	Fruit horizontal diameter	TP	Total phosphorus content
FSL	Fruit stalk length	AP	Available phosphorus content
FSI	Fruit shape index	TK	Total potassium content
FPT	Fruit pericarp thickness	AK	Available potassium content
